# Dimeric Structure of the Transmembrane Domain of Glycophorin A in Lipidic and Detergent Environments 

**Published:** 2011

**Authors:** K.S. Mineev, E.V. Bocharov, P.E. Volynsky, M.V. Goncharuk, E.N. Tkach, Ya.S. Ermolyuk, A.A. Schulga, V.V. Chupin, I.V. Maslennikov, R.G. Efremov, A.S. Arseniev

**Affiliations:** Shemyakin and Ovchinnikov Institute of Bioorganic Chemistry, Russian Academy of Sciences

**Keywords:** bitopic membrane proteins, transmembrane domain, dimerization, spatial structure, molecular dynamics, NMR

## Abstract

Specific interactions between transmembrane α-helices, to a large extent, determine the biological function of integral membrane proteins upon normal development and in pathological states of an organism. Various membrane-like media, partially those mimicking the conditions of multicomponent biological membranes, are used to study the structural and thermodynamic features that define the character of oligomerization of transmembrane helical segments. The choice of the composition of the membrane-mimicking medium is conducted in an effort to obtain a biologically relevant conformation of the protein complex and a sample that would be stable enough to allow to perform a series of long-term experiments with its use. In the present work, heteronuclear NMR spectroscopy and molecular dynamics simulations were used to demonstrate that the two most widely used media (detergent DPC micelles and lipid DMPC/DHPC bicelles) enable to perform structural studies of the specific interactions between transmembrane α-helices by the example of dimerizing the transmembrane domain of the bitopic protein glycophorin A. However, a number of peculiarities place lipid bicelles closer to natural lipid bilayers in terms of their physical properties.

## INTRODUCTION 

Membrane proteins constitute more than 25% of the proteome [1], fulfilling some important functions; they ensure the uniqueness of the biological role of each cell membrane and determine its physicochemical properties. The most important cell processes, such as intercellular reception and communication, molecular and ion transport, membrane fusion, etc., are directly associated with the participation of membrane proteins. Interaction between the transmembrane (TM) domains of proteins that are capable of oligomerizing in the membrane is, in many cases, important for the manifestation of their activity. The so-called bitopic membrane proteins, which have a single TM α-helical segment, play the key role in numerous biological processes taking place in the human organism. The regulation of the activity of bitopic proteins in most cases is associated with homo- or hetero-dimerization in the cell membrane, with the active participation of their TM domains [2, 3]. This class of proteins comprises the majority of receptor protein kinases, immunoreceptors, and apoptosis proteins, which play a direct part in controlling the development and homeostasis of all organism tissues, both in the normal and pathological states.

In order to study the physical parameters of the interaction between the TM domains of proteins using solution NMR spectroscopy, it is necessary to place them into a medium that can mimic the cell membrane [4]. To record high-quality NMR spectra, the particles in this medium have to be relatively small, while the sample’s stability needs to permit the performance of a series of long-term experiments. Meanwhile, the choice of the composition of the membrane-like medium, in which the TM-protein complex would have a biologically significant conformation, is very important. Two classes of membrane mimetics are currently in wide use: detergent micelles with a spherical shape and phospholipid bicelles, which are believed to be disc-shaped [5].

The present work is the first comparative study in the world devoted to the investigation of the effect of various membrane-like media on the conformation of the dimerizing TM domain of bitopic protein. Glycophorin A, an antigen-presenting protein on the surface of human erythrocytes, has been widely used as a model object for fine-tuning experimental and theoretical procedures in the study of the spatial structure and intramolecular dynamics of the interacting TM domains of bitopic proteins. The spatial structure of the TM dimer was first determined for the GpAtm domain solubilized in micelles. However, a number of uncertainties in its structure still remain [6–8]. In order to assess the degree to which the membrane-like environment influences the conformation of interacting TM α-helices, the structural and dynamic characteristics of the homodimer of the GpA _61–98_ TM fragment (GpAtm) were investigated in the following two media: detergent dodecylphosphocholine (DPC) micelles and lipid bicelles consisting of a mixture of dimyristoylphosphatidylcholine /dihexanoylphosphatidylcholine (DMPC/DHPC).

## EXPERIMENTAL 

**Preparation of NMR samples of the recombinant GpA _61–98 _ TM fragment (GpAtm) in membrane-like media**

The recombinant peptide corresponding to the fragment R ^61^ VQLAHHFSEPEITLIIFGVMAGVIGTILL-ISYGIRRL ^98 ^ of human GpA (GpAtm), comprising the TM domain (underlined), was prepared following the procedure in [9, 10]. In NMR studies, detergent DPC micelles and small DMPC/DHPC bicelles with a molar ratio of lipids of 1 : 4 were used as membrane-like media. A completely deuterated detergent *d*_38_ -DPC (Cambridge Isotope Laboratories, Andover, MA, United States) and lipids *d*_54_ -DMPC and *d*_22_ -DHPC with deuterized acyl chains synthesized in accordance with [11] were used. Dry powders of the protein and detergents, or lipids, were dissolved in a 1 : 1 (v/v) trifluoroethanol–water mixture and lyophilized. The dry powder was dissolved in a buffer containing deuterated sodium acetate (20 mM, pH 5.0, 5% D _2_ O), EDTA (1 mM) and sodium azide (0.05 mM). Then, five freeze-heat cycles (to ~40°С) followed by suspension in an ultrasonic bath were performed until the solution became completely transparent. All samples were prepared based on 2 mM of мМ GpAtm in 0.5 ml of a micelle or bicelle solution with a molar ratio of peptide/detergent or peptides approximately 1 : 35, providing approximately two molecules of the TM peptide per micelle/bicelle (meanwhile, taking into account the critical micelle concentration of lipids, the effective ratio between the amount of long and short lipids in a bicelle q ≈ 0.3). In each of the two membrane-like media, three GpAtm dimer samples were prepared: using only ^15^ N-labeled or ^15^ N/ ^13^ C-labeled TM peptide and the 1 : 1 mixture of  ^15^ N/ ^13^ C-labeled and non-labeled TM peptide (“isotopic-heterodimer sample”).

The sizes of micelles and bicelles with embedded GpAtm, as well as its secondary structure, were controlled using optical methods, such as dynamic light scattering and circular dichroism. Dynamic light scattering experiments were performed on a DynaPro Titan instrument (Wyatt Technology Corporation, United States) in a 12 µl cell at a temperature of 30°C. The circular dichroism spectra for GpAtm embedded into micelles, bicelles, or liposomes (phospholipid bilayer) were ascertained on a J-810 spectropolarimeter (Jasco, Japan) in a 0.1 mm quartz cell at 30°C and with a peptide concentration of 1 mg/ml. The circular dichroism spectra were analyzed using CDSSTTR software [12]. In order to prepare small monolayer vesicles, a liposome-containing slurry from DMPC at a 1 : 50 peptide/lipid ratio was treated with ultrasound in ice using an ultrasonic disintegrator with a titanium tip (VirSonic-600, United States) until the sample became completely transparent (approximately 10 min).

**NMR spectroscopy, calculation, and relaxation of the spatial structure of the dimer of a GpA _61–98 _ TM fragment solubilized in membrane-like media**

The NMR spectra of GpAtm solubilized in DPC micelles and DMPC/DHPC bicelles at pH 5.0 and 40°С were recorded on UNITY (Varian, Palo Alto, CA, United States) and AVANCE-III (Bruker BioSpin, Rheinstetten, Germany) spectrometers with proton operating frequencies of 600 MHz. The NMR spectra were analyzed using CARA software [13]. Assignment of the ^1^ H-, ^13^ C-, and ^15^ N resonances of the peptide and obtainment of the structural data were carried out using the standard procedure, employing the triple resonance experiments in [14, 15]. The data concerning the intra-molecular dynamics of the TM peptide were obtained by analyzing the ^15^ N relaxation data: the values of the hetero-nuclear ^15^ N{ ^1^ H} NOE, longitudinal ( *Т*_1_ ) and transversal ( *Т*_2_ ) relaxation times, and the effective rotational correlation times (τ _R_ ) were measured in accordance with the procedure described in [16]. The rates of exchange of amide protons for the deuterium of the solvent were estimated from the changes in signal intensities in the ^1^ H/ ^15^ N-HSQC spectral array; the spectra were sequentially collected during a 24 h period for the GpAtm samples that were preliminarily embedded into micelles and bicelles, lyophilized, and then dissolved in D _2_ O.

The spatial structure was calculated in accordance with the standard procedure in [14], utilizing CYANA 3.0 software [17] and using the method of molecular dynamics in torsion angle space and the simulated annealing algorithm. The restraints on interproton intramonomer distances were obtained from the NOE cross peak volumes in the ^1^ H/ ^15^ N-NOESY-HSQC and  ^1^ H/ ^13^ C-NOESY-HSQC spectra accumulated during a mixing time *t*_m _ = 80 ms. Intermolecular NOE contacts at the GpAtm dimerization interface were obtained from the 3D ^1^ H/ ^15^ N/ ^13^ C-F1-filtered/F3-separated-NOESY-HSQC spectra ( *t*_m _ = 80 ms) using the “isotopic-heterodimer sample” [15]. The ranges of the dihedral angles φ, ψ, and χ ^1 ^ of the protein backbone were estimated from the values ^1^ H, ^15^ N, and  ^13^ C of the chemical shifts of the NH-, CαH-, and CO groups of GpAtm in TALOS software [18]. The restraints on hydrogen bonds were added after preliminary calculation of the structure based on the analysis of the data on the rates of amide protein exchange for the deuterium of the solvent and the spatial proximity of amide proteins to the oxygen atoms of the GpAtm backbone in the preliminary array of structures. The restraints introduced were as follows: on angles 140° < NHO < 180° and 130° < COH < 170° and on distances 1.9 Å ≤ d(O, H ^N^ ) ≤ 2.3 Å, 3.0 Å ≤ d(O, N) ≤ 3.4 Å, 3.2 Å ≤ d(C, H ^N^ ) ≤ 3.6 Å [19]. As a result, based on the upper limits on interprotonic distances (taking into account the stereospecific assignment of peptide groups) and the restraints both on the dihedral angles φ, ψ, and χ ^1 ^ and on hydrogen bonds, we calculated the arrays consisting of 200 structures for GpAtm embedded into micelles or bicelles. Then, 20 structures with the lowest values of the penalty function were selected from these arrays in order to be used as representative ones.

The energy relaxation of representative NMR structures of the GpAtm dimer was performed in the explicitly specified hydrated DPC micelles (60 molecules) or in the DMPC bilayer (512 molecules), respectively, by the molecular dynamics (MD) method using the GROMACS 3.3.1 software package [20] as was earlier described in [21]. After balancing and minimizing the energy of the system, MD trajectories with a duration of 2ns and fixed position of GpAtm dimer atoms were calculated. Next, the calculation of MD trajectories with a duration of 10ns and experimental NMR restraints on distances were performed, followed by the ones with 10ns duration, without any restraints (in order to assess the system’s stability).

The CYANA 3.0, MOLMOL [22], and PYMOL [23] software programs were used to analyze and visualize the spatial structures. The hydrophobic properties of the surface of α-helices were calculated using the molecular hydrophobic potential (MHP) approach [24]. The area of contact surfaces between the α-helices was calculated using the DSSP software [25] as the differential between the surface of GpAtm residues that is accessible to the solvent in the monomeric and dimeric states, respectively.

## Results and discussion 

**Spatial structure and intramolecular mobility of the GpAtm dimer**

**Fig. 1:**
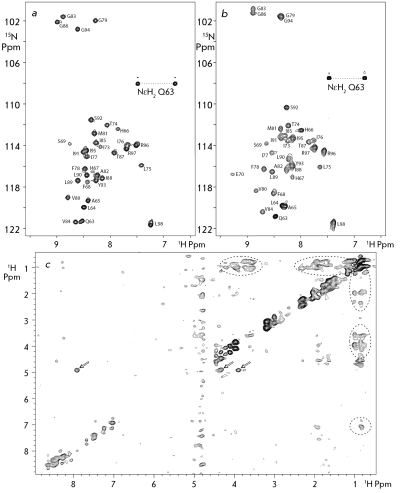
*a* and *b* – Heteronuclear NMR spectrum ^1^ H- ^15^ N HSQC of ^15^ N-labeled GpAtm in DPC micelles and DMPC/DHPC (1/4) bicelles, respectively, with a molar peptide/protein ratio of 1:35, at 40°C and pH 5.0. The resonance assignments are shown. *c* – The intermolecular proton-proton NOE contacts are presented on 2D project of the 3D ^15^ N, ^ 13^ C F1-filtered/F3-edited-NOESY spectrum acquired for the “isotopic-heterodimer” GpAtm sample embedded into the DPC micelles. The NOE cross peaks from the side chain hydroxyl ОγН group of T ^87^ are labeled by arrows, revealing the intramolecular hydrogen-bonding of the hydroxyl group with the carbonyl group of G ^83^ in major conformation of the GpAtm dimer.

The effect of the membrane-like environment on the interactions between the helices in the GpAtm homodimer was studied on the example of two media that have been frequently used in NMR spectroscopy of membrane proteins ( *Figs. 1a,b* ): spherical DPC micelles and disc-shaped DMPC/DHPC lipid bicelles (q ≈ 0.3) [4, 5]. In order to eliminate any possible discrepancies connected with the different procedures of collection of the experimental NMR data and calculation of the spatial structure, the earlier obtained spatial structure of the GpA _62-101_ TM-fragment in DPC micelles [6] is not taken into account in this study.

**Fig. 2:**
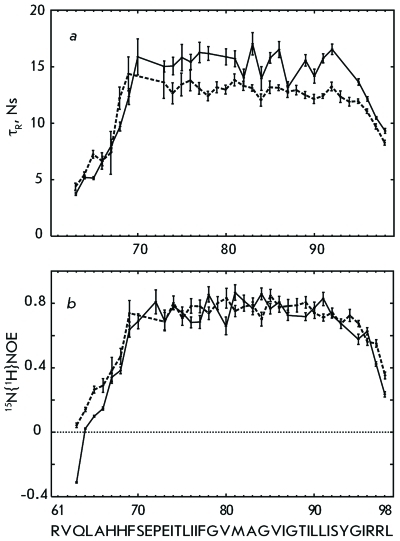
^15^ N relaxation data for the amide groups of the GpAtm dimer embedded into detergent DPC micelles (dashed line) and lipid DMPC/DHPC bicelles (solid line): *a* – effective rotation correlation time *τ*_R_ calculated from the ratio of the ^15^ N longitudinal *T*_1_ and transverse * T*_2_ relaxation times for the corresponding residues; *b* – steady-state ^15^ N{ ^1^ H} NOE values.

The circular dichroism spectra for the GpAtm fragment embedded both into DPC micelles or DMPC/DHPC bicelles and unilamellar DMPC liposomes (phospholipid bilayer) was revealed to be almost identical and as corresponding to an α-helix content of 75 ± 8%. Parameters of the NMR relaxation of the ^15^ N nuclei of the GpAtm backbone: ^15^ N{ ^1^ H} NOE, times *T*_1_ and  *T*_2_ , and calculated effective rotational correlation times (τ _R_ ) of vectors ^15^ N-H ( *Fig. 2* ) attest to the presence of the stable E ^70^ –R ^96 ^ TM segment, which is flanked by flexible N- and C-terminal fragments. The overall rotational correlation time for a peptide/micelle or bicelle complex was estimated from the *T*_1_ / *T*_2 _ ratio at the TM region and is equal to ~13 and ~16 ns. According to the empirical dependence [26], it corresponds to the GpAtm dimer, which forms a complex with ~65 detergent molecules (~34 kDa) or ~70 lipid molecules (~43 kDa). According to the data of dynamic light scattering, both supramolecular systems have a similar hydrodynamic radius of 26 ± 4 Å.

In order to gather information on the interactions between GpAtm helices, we used the NMR spectrum acquired for the “isotopic-heterodimer sample” ( *Fig. 1c* ). This spectrum shows NOE cross peaks corresponding to the magnetization transfer from the protons that are bound to the ^14^ N and  ^12^ C atoms to those bound to the ^15^ N and  ^13^ C atoms [15]. As a result, 17 and 14 intermonomeric NOE contacts were detected in micelles and bicelles, respectively. The sets of intra- and intermonomeric NOE contacts identified in the NMR spectra demonstrated that GpAtm forms a symmetrical, in the NMR time scale, homodimer in both media; this dimer consists of two parallel helices. It should be noted that most of the differences in the systems of NOE contacts observed for GpAtm in micelles and bicelles can be explained via the changes in the chemical shifts of the signals and associated differences in the overlapping of the cross peaks.

**Fig. 3:**
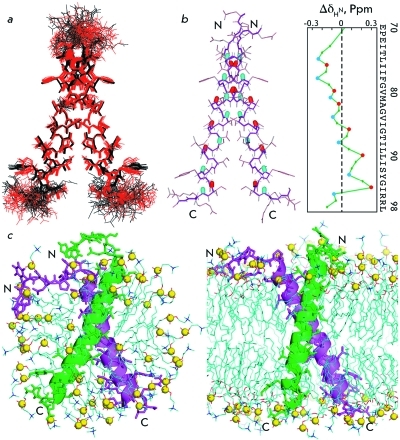
*a* – Comparison of the 20 NMR-derived structures of the GpAtm dimer in DPC micelles (in *black* ) and DMPC/DHPC bicelles (in *red* ) after superposition of the backbone atoms of α-helical residues E ^72^ -I ^95^ of both dimer subunits. The heavy atom bonds of residues (70-98) _2_ are shown. *b* – Differences of the amide chemical shifts (Δδ _HN_ ) of GpAtm in bicelles and micelles are shown on the *right* . The amide groups having local minimal and maximal values of Δδ _HN_ are highlighted in *blue* and *red* , respectively, in the GpAtm dimer structure shown on the *left* . The Δδ _HN_ value strongly depends on the length of the hydrogen bond in which the amide proton participates; thus, the local increase in Δδ _HN_ reflects the shortening of the given hydrogen bond [37]. *c* – Ribbon diagrams of the GpAtm dimer after MD-relaxation in an explicit DPC micelle (on the *left* ) and DMPC bilayer (on the *right* ). *Yellow* balls show the phosphorus atoms of the detergent and lipid heads. Detergent and lipid tails are shown in *blue* . For the sake of clarity, the structures of the adjacent subunits of the dimer are colored in *green* and *magenta* .

The spatial GpAtm structures were determined with high quality and resolution ( *Table, Fig. 3a* ). The experimental NMR restraints that were used to calculate the spatial structures and the resulting atomic coordinates for the arrays of structures of the GpAtm dimer embedded into DPC micelles and DMPC/DHPC bicelles were deposited in the international RCSB data bank of spatial structures (www.rcsb.org); their IDs are 2kpe and 2kpf, respectively. The representative structures of the GpAtm dimer were subjected to energy MD relaxation in an explicitly specified DPC micelle and DMPC lipid bilayer with experimental restraints imposed on distances. This made it possible to adapt the NMR structure to the model membrane environment (Fig. 3c) and determine whether the force fields that are used in the MD calculation correspond to the experimental results. In both cases, the extension of the MD trajectory without restraints did not result in any considerable changes in the dimer structure, which indicates both its relative stability and the admissibility of the force fields used.

In general, the spatial structure and intramolecular mobility of the GpA TM domain in micelles and bicelles differ to an insignificant degree: in both cases, the axes of transmembrane α-helices are located at an angle θ that is equal to -40°, the distance *d * between them being approximately 6.5 Å. Moreover, a comparison of the spatial structures of the dimer ( *Fig. 3a* ) shows that when passing from micelles to bicelles, a small distortion at the C-terminus of the TM-helices occurs. The periodic character of the changes in the chemical shifts of the signals of amide protons (Δδ _HN_ ) along the GpAtm amino acid sequence when passing from micelles to bicelles also points to a small distortion at the C-terminus of the helices ( *Fig. 3b* ). The tendency towards variation of the average values of Δδ _HN _ from negative to positive values along the TM helix points to a possible small stretching of its N-terminal fragment before the dimerization interface and compression, after the N-terminal fragment. Along with the general inclination of the GpAtm dimer with respect to the norm of the lipid DMPC bilayer, a small distortion of the C-termini of TM helices was also observed during MD-relaxation ( *Fig. 3c* ). On the contrary, no considerable distortions of TM helices in the GpAtm dimer, embedded into a DPC micelle, were detected neither in the set of calculated structures, nor during MD relaxation. Since acyl chains are shorter in DPC (formed by 12 carbon atoms) as compared with DMPC (14 carbon atoms), a longer TM helix could be expected (e.g., due to the partial transition of the α-helix into helix 3/10) in bicelles as compared with micelles. However, in the resulting set of NMR structures of the GpAtm dimer in micelles and bicelles, no difference in the length of the α-helix fragments was detected. It was only during MD relaxation in an explicitly specified micelle for the first turn of the TM helix of GpAtm that helix-coil transitions were occasionally observed ( *Fig. 3c* ). This is in agreement with the fact that micelles are more flexible structures in comparison with bicelles and are capable of adapting, to a larger extent, to the shape and size of the TM protein [27, 28]. In turn, micelles compared with bicelles can provide more freedom to conformational dynamics for the TM protein embedded into them. Indeed, during MD relaxation of GpAtm without the imposition of NMR restraints, the dimer parameters were characterized by amplitudes of random fluctuations in DPC micelles (θ 46 ± 6°, *d*  6.3 ± 0.8 Å) that were twice as large as those in the DMPC bilayer (θ 42 ± 3°, *d* 6.4 ± 0.4 Å), which points to a denser packing of the TM helices of the dimer in a lipid environment. The changes in the spatial structure of the GpAtm dimer observed by NMR in bicelles as compared with micelles, and the changes in the course of its MD relaxation in the lipid bilayer, seem to be a result of the adaptation of the dimer to the DMPC bilayer in order to prevent the so-called “hydrophobic mismatch” [29, 30].

**GpAtm dimerization surface**

**Fig. 4:**
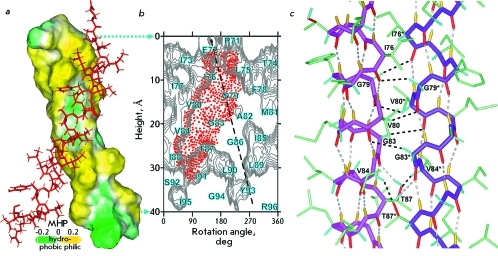
*a* – Hydrophobic and hydrophilic (polar) surfaces of the TM helix of the GpAtm dimer colored in *yellow* and *green* according to the molecular hydrophobicity potential (MHP). The second dimer subunit is presented in *red* . *b* – Hydrophobicity map for the GpAtm helix surface with contour isolines encircling hydrophobic regions with high values of MHP. Details about map construction are described in [21]. The GpAtm helix packing interface is indicated by a red-point area covering the tetrad repeat GG4-like motif G ^79^ VxxG ^83^ VxxT ^87^ employed upon GpAtm self-association in the DPC micelles and DMPC/DHPC bicelles. The potential dimerization heptad repeat motif LI ^76^ xxG ^79^ xxAG ^83^ xxG ^86^ xxLL ^90^ xxY ^93^ inherent to left-hand helix-helix interactions is marked by a *dashed* line. *c* – Central part of the dimerization interface of GpAtm. The intramonomeric and noncanonical Cα—H∙∙∙O intermonomeric hydrogen bonds are shown in gray and black, respectively.

GpAtm helices embedded into a DPC micelle or DMPC/DHPC bicelle associate into a parallel dimer via the so-called tandem four-membered GG4 motif [31] G ^79^ VxxG ^83^ VxxT ^87^ , which is also known as the “glycine zipper” [32]. The motif is formed by the residues with a small side chain, which permits to obtain a tight right-handed packing of the TM helices of GpAtm that contact via their weakly polar surfaces ( *Figs. 4a,b* ) in the hydrophobic environment. Meanwhile, the alternative “seven-membered” motif LI ^76^ xxG ^79^ xxAG ^83^ xxG ^86^ xxLL ^90^ xxY ^93 ^ with left-handed packing of TM α-helices predicted earlier by molecular modelling [33] is not involved.

In both media, there are eight polar intermolecular interactions of Cα—H···O type at the GpAtm dimerization interface. These interactions, which can be characterized as non-canonical hydrogen bonds (with the corresponding distance d(O, H) < 3 Å and angle COH > 120° [34]), are formed between CαH G ^79^ , G ^83^ , V ^80^ , and V ^84 ^ and the carbonyl groups I ^76^ , G ^79^ and V ^80^ , as well as the ОγН-group of T ^87^ , respectively ( *Fig. 4c* ). Quantum chemical calculations demonstrated that the presence of interactions of this type should result in a considerable change in the chemical shifts of the proton signals of the CαH-groups of the proteins [35]. In other words, the chemical shifts of the proton signals of CαH are a very sensitive sensor determining the distances to the carbonyl groups at the dimerization interface of α-helices. The chemical shifts of protons CαH G ^79^ , G ^83^ , V ^80^ , and V ^84 ^ almost completely coincide in micelles and bicelles (the maximum difference is 0.05 ppm), which demonstrates a high degree of identity between the structural organizations of the GpAtm dimerization interface in both media. In other words, both the general topology and structural details of the GpAtm dimerization interface are identical in both membrane-like media.

**Table 333:** Structural statistics for representative ensembles of 20 NMR-derived structures of the GpAtm dimer in the DPC micelles and DMPC/DHPC bicelles

NMR structure	micelle	bicelles
PDB code	2kpe	2kpf
NMR data for structure calculation		
Total unambiguous NOE restraints	484	520
intra-residue	234	278
inter-residue	216	214
sequential (|i-j|=1)	128	128
medium-range (1<|i-j|<4)	88	86
long-range (|i-j|>4)	0	0
inter-monomeric	34	28
Hydrogen bond restraints (upper/lower) intra-monomeric inter-monomeric	108/108 0/0	108/108 0/0
Total torsion angle restraints	156	156
backbone φ	56	56
backbone ψ	56	56
side chain χ^1^	44	44
Structure calculation statistics		
CYANA target function (Å^2^)	0.75±0.15	1.02±0.16
Restraint violations		
distance (>0.2 Å)	0	0
distance (>0.1 Å)	6	5
dihedral (>5^o^)	0	0
Average pairwise RMSD (Å)		
ТМ α-helix (72-95)_2_		
backbone atoms	0.39±0.17	0.42±0.13
all heavy atoms	0.94±0.18	1.07±0.15
generalized RMSD		
backbone atoms	0.72±0.45	
all heavy atoms	1.25±0.37	
backbone atoms of mean structures	1.03	
Ramachandran analysis % residues (70-98)_2_		
in most favored regions	92.7	90.4
in additional allowed regions	7.7	6.4
in generously allowed regions	1.4^†^	0.2^†^
in disallowed regions	0.4^†^	0.7^†^
Helix-helix packing		
helix-helix contact surface (Å^2^)	370±20	380±20
angle*θ*(deg.) between the ТМ helix axes	-40±2	-40±2
distance*d*(Å) between the ТМ helix axes	6.7±0.4	6.4±0.4

Table note:

* Residues from unfolded and flexible regions.

**Comparison of the newly obtained GpAtm structures and those published earlier**

The structures obtained within the framework of our study agree well with the earlier published data on mutagenesis [31]. On the other hand, the GpAtm structure both in micelle and bicelle turned out to be close (RMSD ~ 1.1 Å on the basis of coordinates of the backbone atoms of residues (72–95) _2_ ) to the earlier published structure of the GpA _62-101 _ TM fragment embedded into the DPC micelle [6]. Later, the conformation of the dimer GpA _70-98 _ TM fragment in dried lipid bilayers consisting of DMPC and palmitoyl-oleyl phosphatidylcholine (POPC) was proposed on the basis of the structural restraints obtained using the method of solid-state NMR [7, 8]. In addition to a small decrease in the angle between the axes of the TM helices to -35° and their relative ~25° rotation in the dimer, the character of the hydrogen bond that is formed by the side chain of T ^87 ^ is the main distinction from the structure in a DPC micelle. Based on the proximity of the OγH group of threonine with the carboxyl group V ^84 ^ of the opposite helix, Smith *et al* . [8] arrived at a conclusion that this bond has an intermolecular character. According to the structures of the GpAtm dimer embedded into micelles and bicelles, which was obtained in this study, the distance between the oxygen atoms of the hydroxylic and carbonyl groups of the T ^87^ and V ^84 ^ residues in neighboring monomers is equal to ~4 Å. Meanwhile, in bicelles, these atoms are juxtaposed to ~3.8 Å, which in fact allows this group to form the intermolecular hydrogen bond. However, the system of NOE contacts, which is observed in the NOESY spectra recorded both in bicelles and micelles ( *Fig. 1c* ), unequivocally attests to the fact that in the major conformation of GpAtm, the ОγН group of T ^87 ^ forms an intramolecular bond with the carbonyl group G ^83 ^ (juxtaposed to ~2 Å) ( *Fig. 4c* ). Nevertheless, non-symmetric short-live states with the intermolecular hydrogen bond between the ОγН groups of Т ^87^ (with rotation of the angle χ ^1 ^ of the side chain T ^87 ^ from the gauche(+) into gauche(-) position) were detected in both cases during MD relaxation. A similar effect was detected in other studies devoted to simulating the dimerization of the GpA TM domain [33, 36].

## CONCLUSIONs 

A comparative study of the spatial structure and dynamics in two membrane-like media of different types has been carried out for the first time for specifically interacting TM helices. This significant methodological moment allows one to arrive at the conclusion that in the case of the GpA TM domain, the general topology of the dimer, determined by the specific character of the helix-helix interaction, is independent of the selection of the membrane-like medium; only the local structures of TM helices are to a certain extent sensitive to this factor. On the other hand, it is known that the disc-shape and lipid composition of bicelles brings them closer to a natural lipid membrane in terms of physical properties, which results in a decrease in both the conformational fluctuations of helices and the fluctuations of the parameters characterizing their relative arrangement (angle θ and distance *d* between the helices). In turn, other conditions being equal, this should enhance the stability of the spatial structure of the α-helix membrane protein in bicelles, as compared with that in micelles.
